# CYP2C19 Phenotype, Stent Thrombosis, Myocardial Infarction, and Mortality in Patients with Coronary Stent Placement in a Chinese Population

**DOI:** 10.1371/journal.pone.0059344

**Published:** 2013-03-12

**Authors:** Xiang Xie, Yi-Tong Ma, Yi-Ning Yang, Xiao-Mei Li, Xiang Ma, Zhen-Yan Fu, Ying-Ying Zheng, Bang-Dang Chen, Fen Liu

**Affiliations:** 1 Department of Cardiology, First Affiliated Hospital of Xinjiang Medical University, Urumqi, P.R. China; 2 Xinjiang Key Laboratory of Cardiovascular Disease Research, Urumqi, P.R. China; University of Milan, Italy

## Abstract

**Background:**

Several studies have indicated that CYP2C19 loss-of-function polymorphisms have a higher risk of stent thrombosis (ST) after percutaneous coronary interventions (PCIs). However, this association has not been investigated thoroughly in a Chinese population. In this study, we aimed to determine the effect of CYP2C19*2 and CYP2C19*3 loss-of-function polymorphisms on the occurrence of ST and other adverse clinical events in a Chinese population.

**Methods:**

We designed a cohort study among 1068 consecutive patients undergoing intracoronary stent implantation after preloading with 600 mg of clopidogrel. CYP2C19*2 and CYP2C19*3 were genotyped by using polymerase chain reaction-restriction fragment length polymorphism analysis. The adverse clinical events recorded were ST, death, myocardial infarction (MI), and bleeding events. The primary end point of the study was the incidence of cumulative ST within 1 year after PCI. The secondary end point was other adverse clinical outcomes 1 year after the procedure.

**Results:**

The cumulative 1-year incidence of ST was 0.88% in patients with extensive metabolizers (EMs) (CYP2C19*1/*1 genotype), 4.67% in patients with intermediate metabolizers (IMs) (CYP2C19*1/*2 or *1/*3 genotype), and 10.0% in patients with poor metabolizers (PMs) (CYP2C19*2/*2, *2/*3, or *3/*3 genotype) (*P*<0.001). The one-year event-free survival was 97.8% in patients with EMs, 96.5% in patients with IMs, and 92.0% in patients with PMs (*P* = 0.014). Multivariate analysis confirmed the independent association of CYP2C19 loss-of-function allele carriage with ST (*P* = 0.009) and total mortality (*P*<0.05).

**Conclusion:**

PM patients had an increased risk of ST, death, and MI after coronary stent placement in a Chinese population.

## Introduction

Stent thrombosis (ST) and other adverse clinical events, including myocardial infarction (MI) and bleeding events, are life-threatening complications of percutaneous coronary intervention (PCI). Dual antiplatelet treatment with aspirin and clopidogrel is routinely administered to prevent thrombotic events, including ST and MI, after PCI. However, this therapy significantly increases the risk of bleeding events and related death [1]. Clopidogrel is an inactive prodrug and requires metabolization and activation by the CYP2C19 to generate its active thiol metabolite, which can significantly inhibit platelet aggregation by binding to the ADP P2Y12 receptor [2]. Recent reports suggest that two loss-of-function variants in CYP2C19 are associated with an increased rate of recurrent cardiovascular events, including ST [3]–[5]. These two main enzyme loss-of-function alleles are CYP2C19*2 and CYP2C19*3. CYP2C19*2 is a single base pair G681A mutation in Exon 5 of CYP2C19. CYP2C19*3 is a single base pair G636A mutation in Exon 4 of CYP2C19, which results in a premature stop codon [6], [7].

Sibbing et al. [3] enrolled 2485 consecutive patients undergoing coronary stent placement after pretreatment with 600 mg of clopidogrel and found that the cumulative 30-day incidence of ST was significantly higher in subjects with the CYP2C19*2 allele vs. CYP2C19 wild-type homozygotes (1.5% vs. 0.4%). Harmsze et al. [4] also reported that carriage of the loss-of-function alleles CYP2C19*2 and CYP2C9*3 increases the risk of ST after PCI. In these studies, the enrolled participants were Europeans, whose minor allele frequency (MAF) of CYP2C19*3 was <1%. Therefore, in these two studies, CYP2C19*3 was not found to be associated with the incidence of ST. However, in our previous study, the frequency of CYP2C19*3 was noted to be 6.250% in coronary artery disease (CAD) patients in a Chinese population [5]. Therefore, more attention should be paid to those populations that have a high frequency of CYP2C19*3, especially in China, where there are about 1.3 billion people, which indicates that there are about 50 million people who carry CYP2C19*3. With regard to CYP2C19*2, MAF was significantly higher in Chinese people (28.4%) than in other ethnicities, such as Europeans (15.3%), Sub-Saharan Africans (14.4%), and African-Americans (10.0%), according to a report from the NCBI database (http://www.ncbi.nlm.nih.gov/pubmed/SNP). However, the associations of the CYP2C19 genotypes with the risk of ST and other adverse clinical events after PCI have not been thoroughly investigated.

In this study, we aimed to determine the effect of CYP2C19 loss-of-function polymorphisms on the occurrence of ST and other adverse clinical events in a Chinese population.

## Methods

### Ethics Statement

The present study complies with the Declaration of Helsinki and was approved by the Ethics Committee of the Fist Affiliated Hospital of Xinjiang Medical University. All patients gave written informed consent before study inclusion.

### Patients

**Figure 1:**
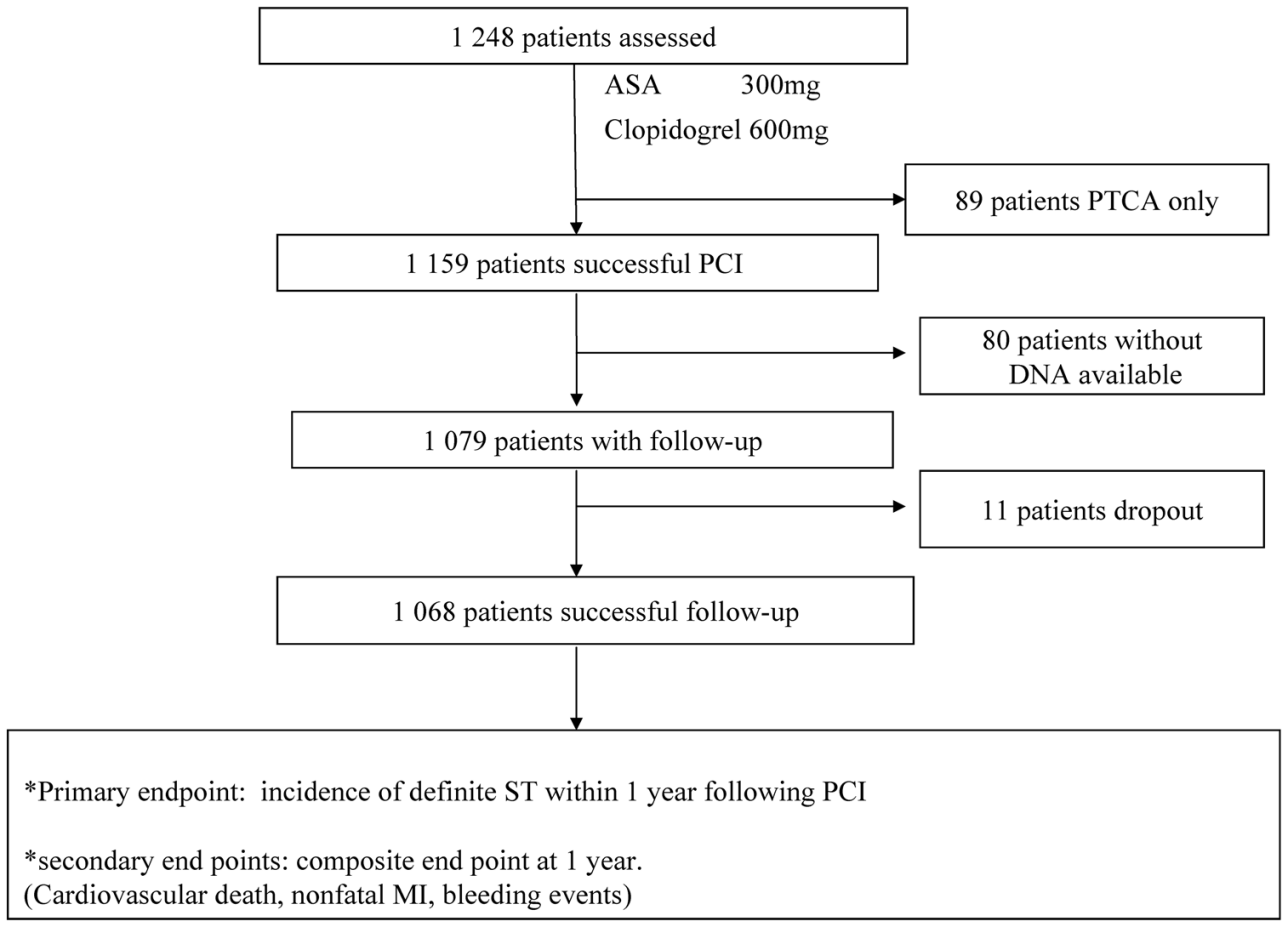
Study flow chart. DNA:desoxy-ribonucleic acid; PTCA, conventional balloon angioplasty.

A total of 1068 patients with CAD undergoing PCI in the First Affiliated Hospital of Xinjiang Medical University from January 2008 to March 2010 were enrolled in the present study. Figure 1 presents the flowchart of the study population. All the patients included in this study were pretreated with a loading dose of 600 mg of clopidogrel for ≥2 h before the procedure. Coronary interventions were done according to the current standard guidelines as described previously [8]. Intravenous anticoagulative treatment with unfractionated heparin was administered to the majority of patients. A small subset of the patients (<10%) received intravenous antiplatelet therapy with glycoprotein IIb/IIIa inhibitor, in addition to a reduced dose of heparin. During the time period after the procedure, the patients were treated and discharged with a dual antiplatelet regimen of 75 mg of clopidogrel (once daily) and 100 mg of aspirin (once daily) for at least 12 months.

The inclusion criterion was clopidogrel-naive patients with CAD, including non–ST-segment elevation acute coronary syndrome (ACS) patients undergoing coronary angiography. The exclusion criteria [3] were as follows: age >75 years, primary PCI for ST-segment elevation acute MI, severe anemia or platelet count <70×10^9^/L, uncoalesced peptic ulcer, high risk of active bleeding, cerebrovascular accident <3 months, history of malignancy, and severe liver disease or chronic renal failure (serum creatinine >2 mg/dL).

Systemic arterial hypertension was defined as a systolic blood pressure (SBP) of ≥140 mm Hg and/or a diastolic blood pressure (DBP) of ≥90 mm Hg on at least two separate occasions, or antihypertensive treatment [9]. Hypercholesterolemia was defined as a documented total cholesterol value of ≥240 mg/dL (≥6.2 mmol/L) or current treatment with cholesterol-lowering medication [9]. The smoking status classifications were current smokers, former smokers, and never-smokers. Alcohol drinking was classified as current drinking, former drinking, and never-drinking. Diabetes mellitus was defined according to the American Diabetes Association (ADA) 2009 criteria [10] (fasting plasma glucose ≥7.0 mmol/L [≥126 mg/dL]) or self-reported current diabetes treatments.

### Biochemical Analysis

Serum and plasma collected for measurement were immediately frozen at −80°C until analysis. We measured the serum concentration of total cholesterol, triglyceride, blood urea nitrogen (BUN), creatinine (Cr), low-density lipoprotein (LDL), high-density lipoprotein (HDL), uric acid, and fasting glucose with the chemical analysis equipment (Dimension AR/AVL Clinical Chemistry System, Newark, NJ, USA) used by the Clinical Laboratory Department of the First Affiliated Hospital of Xinjiang Medical University, as described previously [11], [12].

### Blood sampling and genotyping

Whole blood for genotyping was obtained from the arterial sheath of all patients directly after diagnostic angiography and before PCI. Genomic DNA was extracted from blood leukocytes with the use of a DNA extraction kit [Tiangen Biotech (Beijing) Co. Ltd], according to the manufacturer's instructions. Genotyping was confirmed by polymerase chain reaction (PCR)-restriction fragment length polymorphism (RFLP) analysis, as described previously [5]. To verify our results, we used sequenced genomic DNAs as positive controls in our assays. To control for correct sample handling, genotyping was repeated in 10% of the patients. All the repeated experiments revealed identical results when compared with the initial genotyping.

Individuals can be divided into three groups according to the CYP2C19 genotype. Those who inherit two mutant CYP2C19 alleles (*2 and/or *3) have a reduced capacity to metabolize CYP2C19 substrates and are defined as poor metabolizers (PMs). Individuals who are homozygous (*1/*1) for wild-type CYP2C19*1 have efficient enzymes to metabolize CYP2C19 substrates and are defined as extensive metabolizers (EMs). Subjects who are heterozygous (*1/*2, *1/*3) for wild-type CYP2C19*1 are defined as intermediate metabolizers (IMs) [13]–[15]. Patients who carry a loss-of-function CYP2C19 allele have 1.53- to 3.69-fold increased risk of major cardiovascular events compared with noncarriers [16], [17].

### Study end points and definitions

The primary end point of this study was the cumulative incidence of ST during a 1-year follow-up period. The secondary end point was the other adverse clinical outcomes, including death, MI, and bleeding events, 1 year after the procedure. We defined ST according to the Academic Research Consortium (ARC) 2007 criteria [18] and classified it by the level of certainty (definite, probable, or possible) and the timing of the event (early [0–30 days] or late [31 days to 1 year]). Definite ST was defined as an angiographically or pathologically confirmed thrombus, along with ischemic symptoms or signs. Probable ST was defined as any unexplained deaths within 30 days or acute MI of the target vessel territory without angiographic evidence. Possible ST included any unexplained deaths after more than 30 days. In the present study, we defined the cumulative incidence of ST, including these three categories. MI was defined as new Q waves and an increase in the creatine kinase MB concentration to greater than five times the upper limit of the normal range, if occurring within 48 h after the procedure, or as new Q waves or an increase in creatine kinase MB concentration to greater than the upper limit of the normal range, plus ischemic symptoms or signs, if occurring >48 h after the procedure, as described previously [19]. Bleeding events were defined according to the Bleeding Academic Research Consortium (BARC) criteria [20]. All the events were adjudicated by an event adjudication committee blinded to the genotype of the patients.

### Follow-up

All the patients stayed in hospital for at least 48 h after inclusion and PCI. The patients were interviewed after 30 days (±3 days) and 1 year (±7 days), respectively. All the patients were recommended to have a follow-up coronary angiography in 9 months. The investigators followed the patients either by office visits or telephone calls as necessary. Compliance of the drugs and adverse events were assessed at every visit for clinical follow-up. Those patients with cardiac symptoms received complete clinical, electrocardiographic, and laboratory check-up in the outpatient clinic. A standardized questionnaire was used in the present study to collect information on each subject's medical history, medication status, and lifestyle characteristics.

### Statistical Analysis

All analyses were carried out using SPSS version 17.0 (SPSS Inc., Chicago, IL, USA). The Hardy-Weinberg equilibrium was assessed using chi-square analysis. Discrete variables, expressed as counts or percentages, were compared by chi-square or Fisher's exact test, as appropriate. Continuous variables were expressed as mean ± standard deviation (SD). Normally distributed continuous variables were compared by *t*-test, and non-normally distributed data were compared by nonparametric test. The independent association between the presence of the CYP2C19*2 or *3 allele and its outcome was assessed after adjusting for other potential confounding factors by using multivariate Cox regression analysis and a Cox proportional hazards model for event-free survival. All variables associated with *P*<0.1 in the univariate analysis were entered into the multivariate model as covariates. A value of *P*<0.05 was considered as statistically significant.

## Results

### Patient Characteristics and CYP2C19 Genotype

Among the 1068 patients, there were 524 (49.06%) with wild homozygous, 465 (43.54%) with heterozygous, and 79 (7.40%) with mutant homozygous CYP2C19*2, and there were 947 (88.67%) with wild homozygous, 100 (9.36%) with heterozygous, and 11 (1.97%) with mutant homozygous CYP2C19*3. We divided these 1068 patients into three phenotypes based on CYP2C19*2 and CYP2C19*3 genotypes. Accordingly, there were 454 (42.51%) EMs, 514 (48.13%) IMs, and 100 (9.36%) PMs in the present study. Table 1 and Table 2 describe the baseline characteristics of the studied population according to CYP2C19 phenotype. All these variables were well balanced among these three groups (all *P*>0.05). Table 3 shows the angiographic and procedural characteristics, which were also well balanced among the three groups (all *P*>0.05).

**Table 1:** Baseline characteristics of the study population.

Variables	Overall (n = 1068)	Genotypes			P value
		EMs (n = 454)	IMs (n = 514)	PMs (n = 100)	
Age (year)	59.46±11.04	59.44±11.35	59.58±10.85	59.02±10.71	0.897
BMI (Kg/m^2^)	25.93±6.20	25.53±3.72	26.25±3.72	26.05±4.11	0.202
SBP (mmHg)	135.94±26.34	138.00±27.79	134.89±25.46	131.65±23.05	0.074
DBP (mmHg)	83.50±16.53	84.63±17.63	83.11±14.97	79.97±19.08	0.060
Pulse (beats/min)	73.53±10.89	73.49±11.51	73.42±10.26	74.22±11.14	0.803
BUN (mmol/L)	5.15±1.98	5.23±1.90	5.1±2.1	5.06±1.78	0.577
Cr (mmol/L)	79.41±21.13	79.19±21.73	80.40±20.29	75.25±22.37	0.091
URIC (mmol/L)	325.63±89.32	327.39±94.86	327.45±83.29	308.77±93.19	0.154
GLU (mmol/L)	6.22±2.50	6.32±2.49	6.16±2.57	6.14±2.19	0.598
HbAlc (mmol/L)	2.34±3.79	2.2±0.65	2.5±5.41	2.08±0.47	0.411
TG (mmol/L)	2.06±1.62	2.0±1.42	2.09±1.76	2.11±1.68	0.694
TC (mmol/L)	4.23±1.13	4.23±1.21	4.25±1.07	4.13±0.98	0.640
HDLC (mmol/L)	1.06±0.36	1.05±0.3	1.07±0.41	1.07±0.25	0.511
LDLC (mmol/L)	2.44±.940	2.5±0.96	2.42±0.93	2.34±0.88	0.231
APOA (mmol/L)	1.20±.32	1.18±0.31	1.21±0.35	1.17±0.23	0.242
APOB (mmol/L)	0.98±3.11	0.86±0.42	1.1±0.405	0.87±0.234	0.513

**Table 2:** Baseline characteristics of the study population.

Variables	Overall (n = 1068)	Phenotypes			P value
		EMs (n = 454)	IMs (n = 514)	PMs (n = 100)	
Sex, Female, n (%)	854 (20.0)	95 (20.9)	98 (19.07)	21 (21.0)	0.747
Smoking, n (%)					
Never smoking	456 (42.7)	184 (40.52)	226 (43.97)	46 (46.0)	0.779
Former smoking	26 (2.4)	11 (2.42)	13 (2.53)	2 (2.0)	
Current smoking	586 (54.9)	259 (57.05)	275 (53.50)	52 (52.0)	
Drinking, n (%)					
Never drinking	654 (61.2)	283 (62.33)	302 (58.75)	69 (69.00)	0.080
Former drinking	361 (33.8)	152 (33.48)	186 (36.19)	23 (23.00)	
Current drinking	53 (5.0)	19 (4.19)	26 (5.06)	8 (8.0)	
DM, n (%)	330 (30.90)	154 (33.92)	148 (28.79)	28 (28.0)	0.177
Hp, n (%)	647 (60.6)	284 (62.56)	309 (60.11)	54 (54.00)	0.323
Obesity, n (%)	236 (22.1)	91 (20.0)	122 (23.74)	23 (23.00)	0.375
Hypertriglyceridemia, n (%)	473 (44.3)	193 (42.51)	234 (45.53)	46 (36.0)	0.601
Hypercholesterolemia, n (%)	178 (16.7)	76 (16.7)	87 (16.9)	15 (15.0)	0.893

**Table 3:** Angiographic and procedural characteristics.

Variables	Phenotypes			P value
	EMs (n = 454)	IMs (n = 514)	PMs (n = 100)	
Target vessel, n (%)				
Left main	19 (4.19)	27 (5.25)	3 (3.00)	0.362
LAD	325 (71.59)	336 (65.37)	70 (70.00)	
LCX	59 (13.00)	73 (14.20)	11(11.00)	
RCA	51 (11.23)	78 (15.18)	16 (16.00)	
Stent type, n (%)				
Taxus®	70 (15.42)	75 (14.59)	12 (12.00)	0.247
Firebird®	86 (18.94)	111 (21.60)	20 (20.00)	
EXCEL®	37 (8.15)	36 (7.00)	13 (13.00)	
Cypher®	127 (27.97)	132 (25.68)	24 (24.00)	
Partner®	74 (16.30)	68 (13.23)	15 (15.00)	
Endeaver®	29 (6.39)	28 (5.45)	4 (4.00)	
Others	31 (6.83)	64 (12.45)	12 (12.00)	

### Genotyping and outcomes

Table 4 shows the characteristics of the CYP2C19 genotype. After 1 year of follow-up, the total cumulative incidence of ST, death, MI, and bleeding events was 3.56% (38 of 1068), 3.37% (36 of 1068), 4.49% (48 of 1068), and 1.12% (12 of 1068), respectively. The cumulative incidence of ST, death, MI, and total adverse events was significantly higher in PMs than in EMs (10.0% vs. 0.88%, 8.0% vs. 2.2%, 15.0% vs. 2.28%, and 16.0% vs.3.52%, respectively). However, there was no difference in the incidence of bleeding events among the three phenotypes (*P* = 0.187; Table 5).

**Table 4:** Distribution of genotypes of CYP2C19.

Subjects	EMs (n, %)	IMs (n, %)		PMs (n, %)		
	*1/*1	*1/*2	*1/*3	*2/*2	*2/*3	*3/*3
Total	454 (42.51)	493 (46.16)	21 (1.97)	81 (7.58)	8 (0.75)	11 (1.03)
Men	359 (33.61)	400 (37.45)	16 (1.50)	65 (6.10)	6 (0.56)	8 (0.75)
Women	95 (8.90)	93 (8.70)	5 (0.47)	16 (1.50)	2 (0.19)	3 (0.28)

**Table 5:** Accumulated Major Adverse Events During the One-year Period After Intervention.

Clinical outcomes	Phenotypes			*P* value
	EMs (n = 454)	IMs (n = 514)	PMs (n = 100)	
ST, n (%)	4 (0.88)	24 (4.67)	10 (10.00)	<0.001
Death, n (%)	10 (2.20)	18 (3.50)	8(8.00)	0.014
MI, n (%)	13 (2.86)	20 (3.89)	15 (15.00)	<0.001
Any of the above events, n (%)	26 (3.52)	50 (9.73)	16 (16.00)	<0.001
Bleeding events, n (%)	8 (1.76)	4 (0.78)	0 (0)	0.187

**Figure 2:**
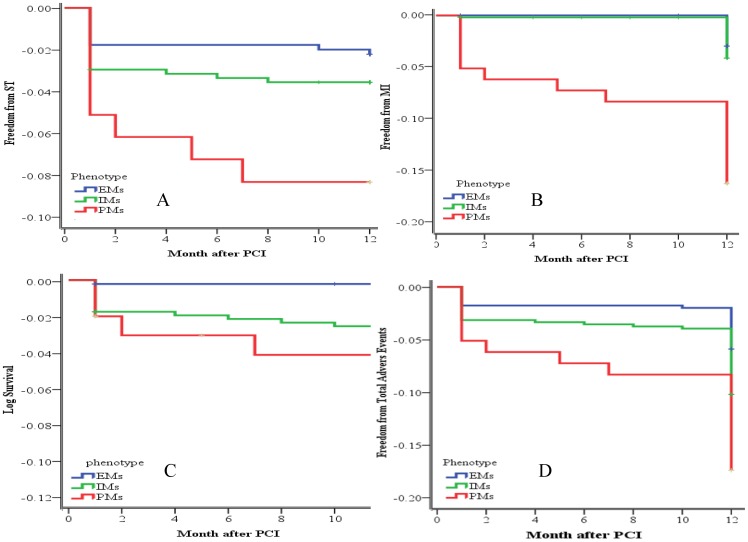
Kaplan – Meier Curves for Event free Survival According to CYP2C19 Loss-of-Function Allele Carrier Status among Chinese Patients with CAD following PCI.

The results of the multivariate Cox proportional hazards model showed that PM carriers were an independent predictor of 1-year ST (HR = 5.268, 95% CI = 1.528–18.164, *P* = 0.009). Table 6 shows the detailed results of the multivariate analysis. There was a decreased adverse-event–free survival rate (including ST, MI, death, and total adverse events) among patients carrying the CYP2C19 PM phenotypes when compared with the EMs (Fig. 2).

**Table 6:** Results of a multivariable Cox proportional hazards model.

Variables	B	SE	P value	Hazard ratio (95.0% CI)
Sex	0.247	0.379	0.514	1.280 (0.609–2.690)
Age	0.052	0.016	0.001	1.054 (1.021–1.087)
Stent type	0.701	0.377	0.063	2.015 (0.963–4.217)
DM	0.299	0.359	0.406	1.348 (0.666–2.727)
HP	0.704	0.394	0.074	2.021 (0.934–4.374)
Hypertriglyceridemia	0.272	0.357	0.447	1.312 (0.651–2.645)
Hypercholesterolemia	0.565	0.415	0.173	1.759 (0.781–3.964)
Phenotype			0.019	
IMs	0.818	0.563	0.146	2.266 (0.751–6.834)
PMs	1.662	0.632	0.009	5.268 (1.528–18.164)

## Discussion

In the present study, we reported the associations of CYP2C19 loss-of-function polymorphisms with the incidences of adverse outcomes of CAD patients after PCI. We found that patients with PMs have much higher incidences of ST, MI, and death when compared with EMs of CYP2C19.

Several studies have provided evidences linking CYP2C19 genetic variation to reduced exposure to the active drug metabolite, lower platelet inhibition, and less protection from recurrent ischemic events in people receiving clopidogrel [21]. According to previous reports, common polymorphisms in the CYP2C19 gene were approximately 30% in whites, 40% in blacks, and >55% in East Asians [22]. As described earlier, according to CYP2C19 genotype, individuals can be divided into three groups: PMs, IMs, and EMs. Our results indicate that PMs accounted for 9.36% and IMs for 48.12% of the Chinese CAD patients. Our result is in line with that reported in previous studies [22].

Although several studies have shown that CYP2C19*2 and CYP2C19*3 polymorphisms influence the platelet response to clopidogrel [23]–[28], clinical data concerning the relevance of CYP2C19*2 and CYP2C19*3 carrier status in patients with CAD after PCI are limited, especially in China. Tang et al. [29] observed 267 CAD patients with PCI. After 1 year of follow-up, the researchers did not find an increased risk of ST among patients with CYP2C19*2, although they observed that the incidence of combined end point was higher in patients with CYP2C19*2 than in patients with CYP2C19*1/*1. Luo et al. [30] reported the relationship between CYP2C19*2 and the incidence of 180-day ST in Chinese Han and found the presence of at least one CYP2C19*2 allele, which was significantly associated with increased ST risk (CYP2C19*2/*2 or *1/*2 patients [2.4%] vs. CYP2C19*1/*1 patients [0.75%]). The risk of definite ST was highest in patients with the CYP2C19*2/*2 genotype. Chen et al. [31] reported that the homozygous CYP2C19*2/*2 genotype is an independent determinant of adverse vascular events in Chinese patients with CAD (HR = 5.191; 95% CI = 1.936–13.917; *P* = 0.001). Oh et al. [32] also reported that the CYP2C19*2 genetic variant may be associated with worse outcome in Korean patients treated exclusively with DES and dual-antiplatelet therapy due to a significant increase in cardiac death, MI, or ST. However, Galiavich et al. [33] found that CYP2C19 gene polymorphism does not influence the prognosis for the next 6 months if patients follow medical recommendations, including regular use of clopidogrel. In their studies, they only selected CYP2C19*2 for examination. Although Tazaki et al. [34] selected not only CYP2C19*2 but also CYP2C19*3, they only described that a test can rapidly detect CYP2C19 PMs and predict low responders to clopidogrel. In addition, Tello-Montoliu et al. [35] examined CYP2C19*2 and *17 and found that even though the CYP2C19 genotype is associated with a variable on clopidogrel platelet reactivity, it has no significant clinical influence. In our study, we selected two loss-of-function polymorphisms (CYP2C19*2 and CYP2C19*3), observed 1068 CAD patients, and conducted 12-month follow-up. In our analysis, we divided the participants into three groups (EMs, IMs, and PMs). We observed that the PMs have higher incidences of MI, ST, and death compared with the EMs. After adjusting for other confounders, the PMs were found to remain as an independent risk factor for ST. Our finding is not only in line with the work carried out by Sibbing et al. [3] but also consistent with pharmacodynamic [25], [26] and pharmacokinetic [23], [24] investigations showing the strongest attenuation of platelet response to clopidogrel treatment among patients homozygous (*2/*2) for the mutant CYP2C19 allele. In addition, we also found that other clinical end points, such as MI and death, were associated with PMs. This result is not in line with Sibbing et al. and Trenk et al. [27], both of which only examined CYP2C19*2 and compared the clinical outcomes between subjects carrying the CYP2C19*2 allele and those carrying CYP2C19*1/*1. The strength of our study is the selection of two loss-of-function polymorphisms (CYP2C19*2 and CYP2C19*3) and the division of the subjects into three groups, which may assist in finding the association between clinical outcomes and the combined effects of CYP2C19*2/*2, CYP2C19*3/*3, and CYP2C19*2/*3. However, our result is in line with a meta-analysis, which revealed that CYP2C19*2 carriers have not only higher ST incidence (OR = 3.03, *P*<0.001) but also higher cardiovascular mortality (OR = 1.79, *P* = 0.019) [36].

In our results, the total incidence of ST is 3.56%, which appears higher than that in a previous report (0.8–2.0%). Many factors, including diabetes, active smoking, prior or ongoing MI, heart failure, recent cancer, renal insufficiency [37], and angiographic characteristics, such as small arteries, long lesions, bifurcations, thrombotic or ulcerated lesions, or low TIMI flow, influence the prevalence of ST [36]. However, pieces of evidence have been accumulated to suggest that the strongest factor associated with ST is the discontinuation of clopidogrel treatment and CYP2C19 genetic polymorphisms. In coronary patients who are carriers of a genetic variant associated with a loss of function of the CYP2C19 enzyme, the risk of ST on clopidogrel treatment was noted to be 3- to 6-fold higher depending on the population [38], [39], [3], [4], [29], [30], [36], [37]. Our results indicate that after adjustment for other confounders in CAD patients with PMs, the risk of ST increased by 4.268-fold (HR = 5.268; 95% CI = 1.528–18.164).

### Limitation

Due to the absence of some angiographic characteristics, such as vessel diameter, lesion length, and blood flow status, we only included target vessels, stent type, and other clinical characteristics in the multivariate Cox regression model. As a result, overestimation of the effect of CYP2C19 loss-of-function polymorphisms on ST may have occurred.

## Conclusions

PM patients in a Chinese population had an increased risk of ST, death, and MI after coronary stent placement.
